# The Entry Blocker Peptide Produced in *Chlamydomonas reinhardtii* Inhibits Influenza Viral Replication *in vitro*

**DOI:** 10.3389/fpls.2021.641420

**Published:** 2021-05-12

**Authors:** Karen Lizbeth Reyes-Barrera, Ruth Elena Soria-Guerra, Rogelio López-Martínez, Leonor Huerta, Nohemí Salinas-Jazmín, Carlos Cabello-Gutiérrez, Ángel Gabriel Alpuche-Solís

**Affiliations:** ^1^Laboratorio de Biología Molecular de Plantas, División de Biología Molecular, Instituto Potosino de Investigación Científica y Tecnológica A.C., San Luis Potosí, Mexico; ^2^Laboratorio de Biotecnología Molecular de Células Vegetales, Facultad de Ciencias Químicas, Universidad Autónoma de San Luis Potosí, San Luis Potosí, Mexico; ^3^Departamento de Inmunología, Instituto de Investigaciones Biomédicas, Universidad Nacional Autónoma de México, Ciudad de México, Mexico; ^4^Departamento de Farmacología, Facultad de Medicina, Universidad Nacional Autónoma de México, Ciudad de México, Mexico; ^5^Departamento de Investigación en Virología y Micología, Instituto Nacional de Enfermedades Respiratorias “Ismael Cosío Villegas”, Ciudad de México, Mexico

**Keywords:** antiviral peptide, microalgae, nuclear expression, biopharma, hemagglutinin

## Abstract

This year, a respiratory virus caused an emergency pandemic alert in health services around the world, showing the need for biotechnological approaches to fight these diseases. The influenza virus is one of the main viral agents that generate pandemic outbreaks. Currently, the majority of co-circulating influenza A virus (IAV) strains are adamantine‐ and oseltamivir-resistant strains, and the challenge is to find new antivirals for more efficient treatments. The antiviral entry blocker (EB) peptide is a promising candidate for blocking the virus entry into cells. The aim of this research was to express the EB peptide in the microalgae *Chlamydomonas reinhardtii* and test its antiviral activity against IAV *in vitro*. The EB peptide nucleotide sequence was introduced into the nuclear genome of microalgae using *Agrobacterium tumefaciens* transformation. The EB peptide amount produced in transformed microalgae was 4.99 ± 0.067% of the total soluble protein. In hemagglutination inhibition assays using influenza A/H1N1 pdm and influenza A H1N1/Virginia/ATCC/2009 strains, we reported that the EB peptide extract from the microalgae showed 100-fold higher efficiency than the EB synthetic peptide. In addition, both the EB peptide extract and synthetic peptide inhibited viral replication in MDCK cells (IC_50_ = 20.7 nM and IC_50_ = 754.4 nM, respectively); however, the EB peptide extract showed a 32-fold higher antiviral effectiveness than the synthetic peptide against influenza A/H1N1 pdm. Extracts from untransformed and transformed microalgae and synthetic peptide did not show cytotoxic effect on MDCK cell monolayers. Thus, *C. reinhardtii* may be a fast, safe, and effective expression platform for production of peptides with significant antiviral activity and can be used as a prophylactic treatment to reduce viral propagation.

## Introduction

Influenza infections are the main cause of respiratory diseases in the world. The morbidity associated with influenza is high and many cases require hospitalization. The World Health Organization (2019) estimates between 290,000 and 650,000 influenza-associated respiratory deaths per year (Iuliano et al., 2018). Currently, the influenza virus is considered a global threat because it can be spread easily and has the ability to affect all age groups. Worldwide, the main co-circulating strains are influenza viruses A H1N1 and A H3N2. These viruses are dangerous and have caused various pandemic events in human history (Webby et al., 2013; Center for Disease Control and Prevention, 2018).

The viruses have evasion mechanisms for the immune system; therefore, new pandemic strains can arise; for this reason, the influenza vaccines need to be redesigned annually and new control strategies are required (Koel et al., 2015). Currently there are two classes of antiviral drugs in order to treat the flu: adamantine derivatives (such as adamantine and rimantadine) and neuraminidase inhibitors (oseltamivir and zanamavir). However, point mutations in neuraminidase and hemagglutinin have been detected in the circulating strain, which confers high resistance to these antiviral drugs (Jones et al., 2006; L’Huillier et al., 2015; World Health Organization, 2019). This event also demonstrates the urgent need for development of new strategies in order to control the influenza virus infection such as use of antiviral peptides.

The entry blocker (EB) peptide is a 20-amino-acid peptide derived from the fibroblast growth factor 4 signal sequence. This peptide exhibits a broad spectrum of antiviral effects against influenza viruses A, which was tested *in vitro* assays (Jones et al., 2006). This peptide has the ability to impede the virus entry into the cell due to the fact that the EB peptide docks to binding pockets of hemagglutinin and diminishes the affinity for sialic acid receptors. Moreover, it has been observed that EB induces virus aggregation and diminished interaction with permissive cells for the virus entry (Jones et al., 2011a,b).

Therefore, the use of the EB peptide is a promising alternative therapy against influenza virus; however, the organic synthesis is expensive; thus, to use new platforms to produce recombinant proteins is an alternative. Currently, the microalga *Chlamydomonas reinhardtii* has stood out as an economic expression system; moreover, the nuclear or chloroplast genetic transformation is relatively fast and stable, and the produced protein shows correct folding and a complex assembly. Additionally, this microalgae is recognized as GRAS (Generally Recognized as Safe) category platform (Mayfield et al., 2003, 2007). The aim of the current work was to express the EB peptide in *C. reinhardtii* microalgae and test its antiviral activity *in vitro* against influenza A virus H1N1.

## Materials and Methods

### Microalgae Strain and Culture Conditions

The strain CC-137 mt (+) of *C. reinhardtii* was transformed with vectors harboring the EB sequences. Microalgae cultures were cultivated in Tris-Acetate-Phosphate medium (TAP) either solid or liquid (120 rpm of orbital agitation) at 23°C with 16/8 h light/dark cycle.

### Synthetic Gene Design and Microalgae Transformation

**Figure 1:**
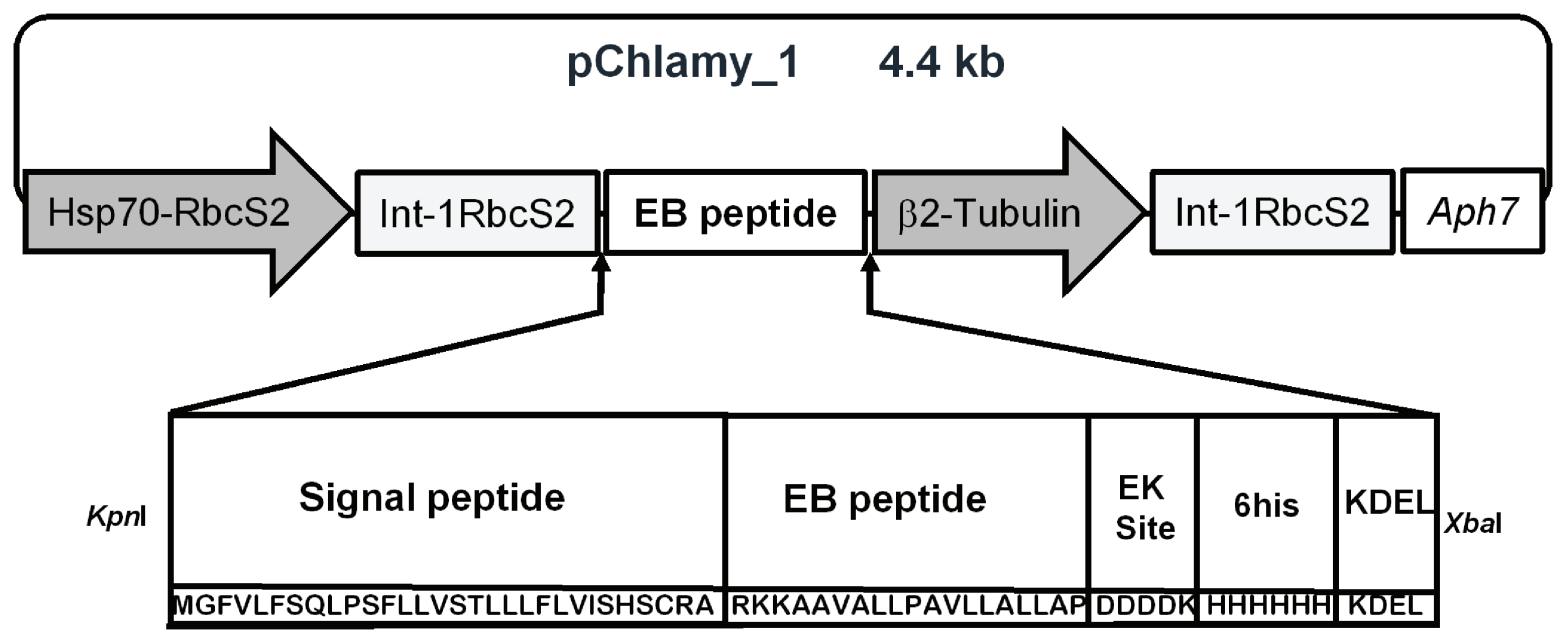
Components of the transformation vector for EB expression in *Chlamydomonas reinhardtii*. EB gene sequence optimized for expression in *Chlamydomonas reinhardtii* cloned between *KpnI* and *XbaI* restriction sites into pChlamy_1 vector. The EB peptide amino acid sequence has additional components: Signal peptide: Pr1a sequence; EK site: Enterokinase protease site; 6his: six-histidine tag; KDEL: reticulum endoplasmic retention tag. The pChlamy vector has the following elements: Hsp70A-RbcS2 promoter, Int-RbcS2 is RuBisCO Small Subunit 2 intron, B2 tubulin promoter, and *Aph*7 gene from hygromycin resistance.

The nucleotide sequence of the EB antiviral peptide was codon optimized for nuclear microalgae expression. The peptide design contains the Pr1a signal peptide (Sharma et al., 2000), an enterokinase protease site, a KDEL retention sequence, and a histidine tag. The EB nucleotide sequence was synthesized by GenScript Inc. (Piscataway, NJ, United States). This sequence was cloned into pChlamy_1 vector using *Xba*I and *Kpn*I restriction sites (Figure 1). This construct was confirmed by PCR assay, restriction analysis, and sequencing.

*Agrobacterium tumefaciens* GV3101 strain was transformed with EB-pChlamy construct by electroporation (25 μF, 2.4 kV, and 200 Ω in BTX 630 ECM Electroporator). The transforming bacteria were grown in LB (Luria Bertani) medium containing ampicillin (100 mg/L). Then, the transformed bacteria were corroborated by PCR.

The *C. reinhardtii* nuclear transformation was achieved by co-cultivation of microalgae with *A. tumefaciens* GV3101 strain, following the protocol described by Kumar et al. (2004). The putative transformants were kept in successive selective rounds in solid TAP medium containing hygromycin (15 mg/L). After several rounds of selection, the resistant clones were grown in TAP liquid medium.

### Transgene Detection by PCR

The DNA was extracted of biomass collected from 15 ml of liquid culture from wild type (WT) and TAP-hygromycin-resistant microalgae according to Newman et al. (1990). The identification of EB transgene into the microalgae genome was confirmed by PCR. For nuclear detection of EB gene, the specific primers F-5' TGCTGCTGTTCCTGGTGAT 3' and R-5'GTGGTGCTTGTCGTCGTC 3' were used. The PCR conditions were as follows: 5 min of denaturalization at 95°C, followed by 30 cycles (95°C for 30 s, 62°C for 30 s, and 72°C for 30 s), and a final cycle of 72°C for 5 min. The PCR products were visualized in 1% agarose gel electrophoresis.

### Proteins Extraction and EB Detection

The transformed line and WT microalgae were grown in 1 L of liquid TAP medium, which was inoculated with a pre-inoculum of 100 ml of culture (O.D.700 = 0.3). After 7 days, the biomass was collected by centrifugation at 10,000 rpm at 4°C and lyophilized. The chlorophyll was removed from the dry biomass by washing with acetone, acetone:methanol (50:50), and methanol. The biomass was suspended in an extraction buffer (Milli Q water or 100 mM Tris-HCl, pH 8) containing 0.1% protease inhibitors cocktail (Sigma Aldrich, St. Louis, MO, United States). The biomass was sonicated at 30% of amplitude (4 cycles, 30 s each), centrifuged at 12,000 rpm for 10 min, and supernatant was collected. The protein concentration was measured at 205 nm using a nanodrop 2000 (Thermo Scientific Waltham, MA, United States) according to Altmann et al. (2009). The extracts were analyzed by Tricine 18% SDS-PAGE and 6 M urea and stained with silver nitrate.

The total soluble protein (TSP) from transformed and WT microalgae were analyzed by Western blot. Twenty micrograms of TSP was resolved in Tricine 18% SDS-PAGE under reducing conditions and transferred to polyvinylidene fluoride in a Turbo Transfer System (Bio-Rad, Hercules, CA, United States). The membrane was blocked with Tris-buffered saline and 0.04% Tween-20 (TBS-T) containing 2% hydrolyzed casein. Subsequently, the membrane was incubated with a mouse anti-EB peptide polyclonal antibody at 1:250 dilution in TBS-T for 2 h. Finally, membrane was incubated with an Anti-Mouse antibody conjugated to Horseradish Peroxidase (HRP; A5420 Sigma-Aldrich, St. Louis, MO, United States) at 1:4000 in TBS-T for 2 h. Specific antibody detection was performed using the PIERCE ECL-Western Blotting Substrate kit following the manufacturer instructions, and the signal was captured in X-ray films (Cytiva Amersham™ Hyperfilm™ ECL, Thermo Fisher Scientific, Waltham, MA, United States).

The content of the EB peptide in transformed and WT microalgae of TSP was determined by indirect ELISA. Microtiter plates were coated with 5 μg of TSP. A synthetic EB peptide (RRKKAAVALLPAVLLALLAP) synthesized by GenScript Inc. (Piscataway, NJ. United States) was used to construct a standard curve (1.9–500 ng). The plate was washed with TBS-T and subsequently blocked with hydrolyzed casein at 2% in TBS-T and incubated with a mouse anti-EB peptide polyclonal antibody at 1:250 dilution. After, wells were incubated an Anti-Mouse Alkaline Phosphatase antibody produced in rabbits (A1902; Sigma Aldrich, St. Louis, MO, United States) at 1:1000 dilution. Colorimetric reaction with 3 mM solution of p-nitrophenyl phosphate was read at 405 nm in íMar™ Microplaque Absorbance Reader (Bio-Rad, Hercules, CA, United States). The data were analyzed for one-way analysis of variance (*p* < 0.001 was considered as a statistically significant difference) with Tukey multiple comparisons test. Statistical analysis and graphics were made in GraphPad Prism 6 Software.

### Cytotoxicity Assays

Madin Darby Canine Kidney cells (MDCK) were routinely cultured at 37°C in a humid 5% CO_2_ atmosphere and grown in Minimum Essential Medium (MEM) supplemented with 10% fetal bovine serum (FBS). A viability standard curve was made using 0 to 1.6 × 10^5^ cells in 96-microwell plates. Cells were incubated with 10 μl of MTT [5 mg/ml 3-(4,5-dimethylthiazol-2-yl) bromide-2,5-diphenyltetrazole in PBS] for 4 h at 37°C with 5% CO_2_. Then, the medium was removed, and the formazan crystals were dissolved with 50 μl of DMSO and read at 490 nm in íMar™ Microplaque Absorbance Reader (Bio-Rad, Hercules, CA, United States).

The cytotoxicity assay for the EB peptide was carried out in 1 × 10^4^ MDCK cells. The cells were incubated for 72 h with medium alone, medium containing the synthetic EB peptide, or transformed WT TSP (0 to 50 μg). Medium containing 4 M urea was used as a positive control of damage to the cell monolayer. Cytotoxic effect was determined after 72 h by the MTT reduction method (van Meerloo et al., 2011). The data were analyzed for two-way analysis of variance (*p* < 0.001 was considered as a statistically significant difference) with Dunnett’s multiple comparisons. Statistical analysis and graphics were made in GraphPad Prism 6 Software.

### Virus Propagation and Infectivity Assays

Influenza pdm A/H1N1 (Zuñiga et al., 2011) and Influenza A H1N1 Virginia/ATCC/2009 strains were propagated in MDCK cell line using MEM medium containing 0.5 μg/ml Trypsin TPCK and 0.3% BSA, at 37°C for 72 h, when cytopathic effect was observed. Subsequently, the supernatant was collected and frozen in liquid nitrogen. Virus titer was determined by hemagglutination assays (Balish et al., 2013).

Virus infectivity was determined by plaque formation assays and tissue culture infectious dose 50% assay (TCID_50_). The plaque assays were done in MDCK cellular monolayer, adding 200 μl per well of 10-fold dilution series of viral stock and it was internalized 1 h at 37°C. Low melting agarose 0.6%, MEM medium, 0.5 μg/ml Trypsin TPCK, and 0.3% BSA were used as an immobilization medium. Plaque formation was monitored under a microscope daily until visible cytopathic effect. Also, the TCID_50_ of viral stock was performed as recommended (Balish et al., 2013).

### Hemagglutination Inhibition Assay

Inhibition of hemagglutination induced by the influenza pdm A/H1N1 and influenza A H1N1 Virginia/ATTC/2009 strains was determined for the EB microalgae extract and the EB synthetic peptide. The EB synthetic peptide and EB expressed in microalgae (concentration from 0 to 4000 nM) were incubated with 64 hemagglutination units (HA) of virus for 1 h at 37°C. The WT extracts (0.02 to 200 μg of protein) were used for control. Subsequently, 50 μl of human red blood cell (RBC) suspension (0.05%) was added and incubated for 1 h at 37°C. Each concentration was tested in triplicate. The percentage of inhibition obtained from three independent assays was analyzed in GraphPad Prism 6 Software. The IC_50_ was determined by a dose-response curve (Kawaoka and Neumann, 2012; Spackman, 2014).

### Viral Inhibition Assay

One hundred TCID_50_ of influenza pdm A/H1N1 virus was mixed with 0 to 4 μM of the synthetic peptide EB and EB expressed in microalgae, for 1 h at 37°C. Additionally, as a control, 0.02 to 200 μg of protein from WT extracts (MQ and Tris) was mixed with the virus for 1 h at 37°C, to demonstrate that microalgae proteins do not have an inhibitory effect on viral replication. Each concentration of EB was analyzed in quadruple. Mixtures were added to MDCK cell monolayers and viral adsorption was allowed for 1 h at 37°C. Then, the inoculum was removed, and cells were washed with PBS and incubated in MEM medium, 0.3% BSA, and 0.5 μg/ml TPCK trypsin. The cytopathic effect was observed at 48 h post-infection. Supernatants were collected and viral particles were determined by hemagglutination. The result from two independent assays was analyzed in GraphPad Prism 6 Software. The IC_50_ for each peptide was determined for a dose-response curve (Kawaoka and Neumann, 2012; Spackman, 2014).

## Results

### Selection of *C. reinhardtii* Transformants and Transgene Detection

**Figure 2:**
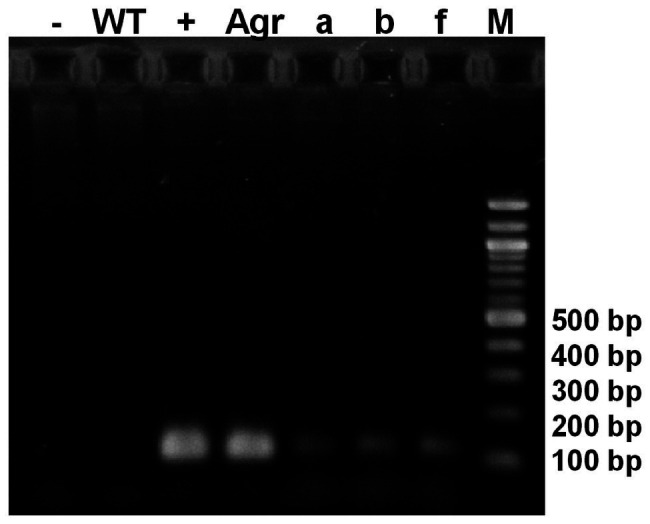
PCR assay for the detection of EB peptide gene in nucleus transformed microalgae. Amplification of an expected 116-bp product corresponding to the EB gene of nuclear transgenic lines (a, b, f), (Agr): Positive control EB-pChlamy_1 purified from *Agrobacterium tumefaciens*; (+): Positive control EB-pChlamy_1 purified from *E. coli*; (−): negative control; (WT): wild type strain DNA; (M) DNA molecular weight marker (100 bp ladder).

The gene encoding the EB peptide was cloned into pChlamy_1 vector and was confirmed by PCR and sequencing analysis. The *C. reinhardtii* was transformed by this construct using *A. tumefaciens*. The 10 putative transformed microalgae were grown in TAP-hygromycin for multiple generations. A 116-bp specific amplicon of EB transgene was detected in the nuclear genome of three resistant microalgae (Figure 2). No change in phenotype characteristics was observed in comparison with the WT microalgae (data not shown). Furthermore, the transgenic line *a* showed stable characteristics after 4,000 generations and was selected for further studies.

### EB Peptide Expression

**Figure 3:**
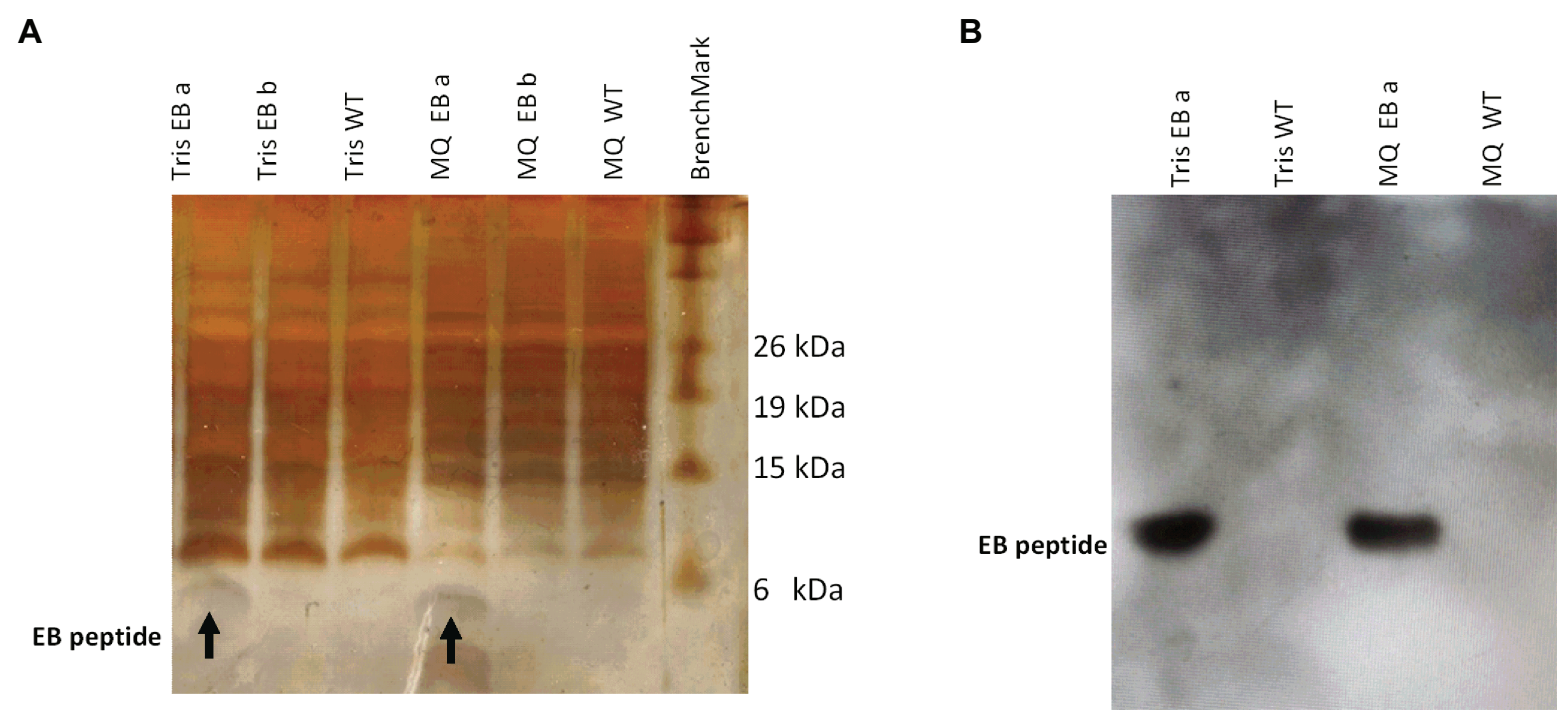
Detection of an estimated 3.4-kDa band in transformed microalgae, corresponding to the EB peptide. **(A)** Protein electrophoresis from transformed and wild-type microalgae. The EB peptide (3.4 kDa estimated size) is observed in Tris and Milli Q water extracts from transformed microalgae lines EB a and EB b, respectively. **(B)** Western blot EB peptide detection with an anti-EB antibody in microalgae extracts. Tris and MQ water EB a line extracts from transformed microalgae are shown as well as Tris and MQ water WT from wild-type microalgae extracts. (MQ): Milli Q water.

It was necessary to remove the chlorophyll from the EB peptide in soluble extract because the chlorophyll inhibits the detection by Western blot. The EB peptide was successfully extracted with Tris and Milli Q water buffers from transformed microalgae. The EB peptide expected band is observed in TSP from transformed microalgae by Tricine SDS-PAGE, showing a 3.4-kDa size, which was the predicted size by bioinformatics programs (ProtParam tool, Expasy); in addition, the EB peptide was not observed in TSP from WT (Figure 3A).

The presence of the EB peptide in the extracts was validated by Western blot using a primary anti-EB peptide polyclonal antibody. Figure 3B shows the 3.4-kDa band from both Milli Q water and Tris-transformed microalgae extracts, while in the WT microalgae extracts, no signal was detected.

**Figure 4:**
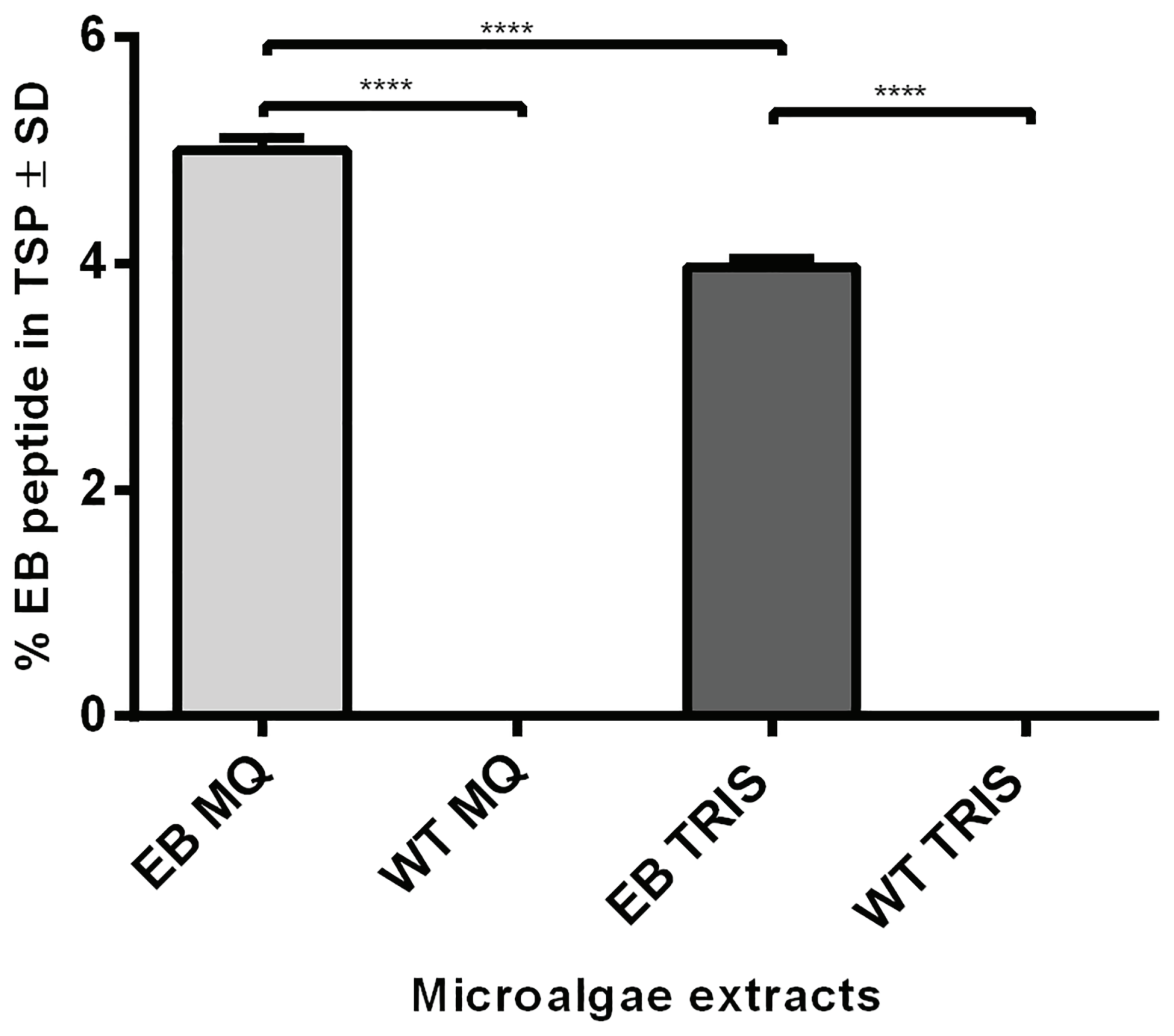
ELISA quantification of EB peptide produced in transformed microalgae. The EB concentration was estimated in transformed (EB) microalgae extracts with a mouse anti-EB peptide polyclonal antibody, using wild-type (WT) microalgae as controls. The amount of EB was expressed in percentages of total soluble protein (TSP; ANOVA one way, *p* < 0.001) with Tukey multiple comparisons test (**** = 0.0001). (MQ): Milli Q water extracts; (TRIS): Tris Buffer extracts.

The EB quantification in transformed microalgae was determined by ELISA (Figure 4); Milli Q water extracts contains 4.99 ± 0.067% of EB in TSP (EB MQ) and TRIS extracts have 3.96 ± 0.046% of EB in TSP (EB TRIS). Regarding the yield of recombinant peptide production, *C. reinhardtii* produce 1.95 mg of EB per gram of dry weight; therefore, this represents 0.19% of dry biomass after chlorophyll extraction.

### Cytotoxicity Assays

**Figure 5:**
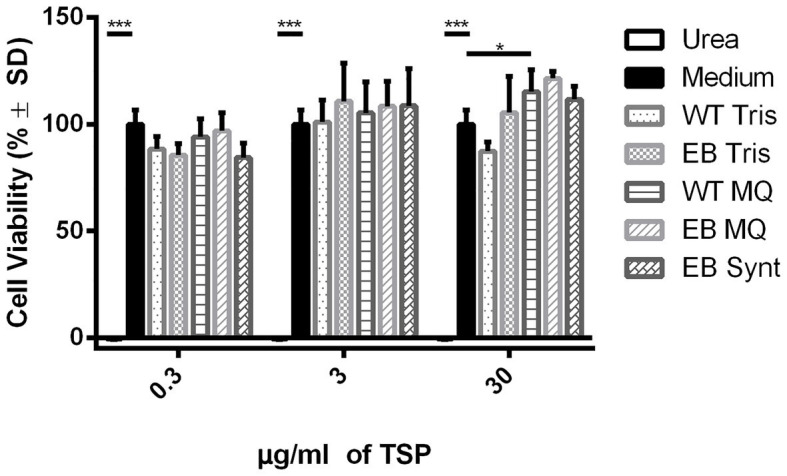
The cell viability is not affected for EB extracts. The EB and WT extracts in Tris and Milli Q water and the EB synthetic peptide (0.03, 3, and 30 μg/ml) did not decrease cellular viability in comparison with medium control (*p* < 0.001, ANOVA). Dunnett’s multiple comparisons test (*** = 0.001; * = 0.1). The % cell viability was determined by MTT reduction. Urea (4 M) was used as a negative control of cell viability reduction, and MEM medium was used as a positive control of cell viability reduction. (MQ): Milli Q water extracts; (TRIS): Tris Buffer extracts.

The peptide cytotoxicity was assayed on MDCK cells by changes in cell viability, using MTT reduction assay. The cell viability did not decrease in the presence of the EB synthetic peptide and the EB or the WT microalgae extracts. These groups had no significant differences with respect to the control (medium alone) in all concentrations tested (0.3, 3, and 30 μg of protein; Figure 5). Consequently, we tested higher concentrations of protein (>30 μg) and neither showed a cytotoxic effect to MDCK cells (data not shown).

### Determination of the Inhibitory Activity of the EB Peptide

**Figure 6:**
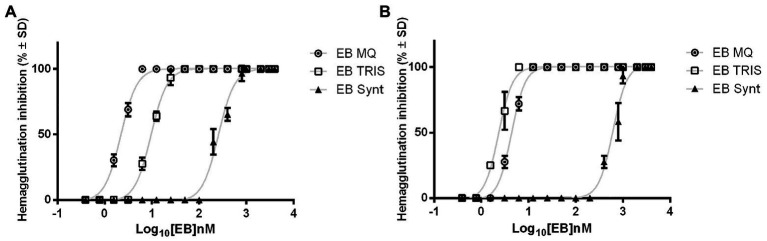
EB blocks the binding of viral hemagglutinin to its receptor. EB expressed in microalgae and synthetic EB inhibited the viral hemagglutination. Dose-response curves of each peptide show the ability of EB to prevent viral hemagglutinin binding to sialic acid in red blood cell membrane. **(A)** EB inhibitory effects vs. influenza pdm A/H1N1 strain. **(B)** EB inhibitory effects vs. influenza A H1N1 Virginia/ATTC/2009 strain. The graph shows the mean hemagglutination inhibition in percentage for each EB ± SD. The results belong to three independent assays. (MQ): Milli Q water extracts; (TRIS): Tris Buffer extracts.

The hemagglutination inhibition test allows one to determine if the EB peptide is capable of binding to the viral hemagglutinin and blocks the interaction with N-acetylmuramic acid of glycoproteins in RBCs. The dose-response curve exhibited that the EB extracts and synthetic peptide can inhibit in a dose-dependent manner the erythrocyte hemagglutination of both influenza pdm and Virginia strains. For the influenza pdm strain, the half maximal inhibitory concentration for binding to receptor was lower for EB expressed in microalgae (2.202 ± 1.027 nM for EB MQ extract and 9.582 ± 1.027 nM for EB Tris extract) than for the EB synthetic peptide (256.3 ± 1.03 nM; Figure 6A; Table 1). This indicates that EB produced in algae is 100-fold more effective than the synthetic peptide. Another interesting fact is that MQ and Tris WT extracts are not able to inhibit the binding between hemagglutinin and its receptor.

**Table 1:** Hemagglutination inhibition of influenza virus by EB peptide.

Virus strains	Sample	IC_50_ (nM) Mean ± SD
A H1N1 pdm	EB MQ extract	2.202 ± 1.027
	WT MQ extract	0
	EB TRIS extract	9.582 ± 1.027
	WT TRIS extract	0
	EB Synthetic	256.3 ± 1.03
A H1N1 Virginia	EB MQ extract	4.388 ± 1.01
	WT MQ extract	0
	EB TRIS extract	2.338 ± 1.032
	WT TRIS extract	0
	EB Synthetic	613.8 ± 1.043

Concentrations from 0 to 4000 nM of EB of transgenic microalgae extracts were analyzed for the ability to inhibit hemagglutination of 64 HA units of virus. The results represent three independent assays. The IC_50_ and SD was determined for the dose-response curve.

In the inhibition hemagglutination assay of influenza Virginia strain, the EB tris extract showed an IC_50_ of 2.338 ± 1.032 nM and EB MQ extract has 4.388 ± 1.01 nM, while the EB synthetic peptide showed an IC_50_ value of 613.8 ± 1.043 nM (Figure 6B; Table 1). Again, the EB peptide expressed in algae is more effective than the synthetically produced peptide against this influenza strain. We observed that WT microalgae extracts do not show the capacity for protection against the sialic acid-hemagglutinin binding.

### The EB Peptide Inhibits Viral Replication

**Figure 7:**
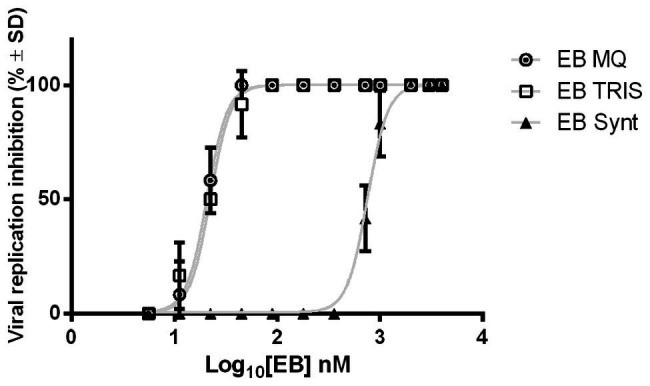
Antiviral effect of EB synthetic and expressed in microalgae. EB expressed in microalgae and synthetic EB can inhibit viral replication of H1N1 A pdm in MDCK cells. Dose-response curves of each peptide show the ability of EB to prevent the replication of 100 TCID_50_ of Influenza pdm A/H1N1. In the graph, mean percentage ± SD of viral replication inhibition for each EB concentration is appreciated. The results belong to two independent assays. (MQ): Milli Q water extracts; (TRIS): Tris Buffer extracts.

The viral replication inhibition assay allows us to assess the ability of EB to inhibit the influenza pdm entry into MDCK cells. The first observation was 100 TCID_50_ of virus, and it caused a strong cytopathic effect that destroyed the entire cell monolayer at 48 h post-infection and produced new viral particles. The dose-response curve exhibits a reduction in viral infection when the EB amount increases (Figure 7). Both EB expressed in microalgae and the EB synthetic peptide inhibited viral replication, but comparing the IC_50_ between peptides, we determined that 36 times less amount of EB extracts (20.71 ± 1.03 nM) than the EB synthetic peptide (754.4 ± 1.02 nM) is necessary to inhibit the viral replication (Table 2). It can be concluded that EB prevents virus replication because we did not detect new viral particles (by hemagglutination assay) in the supernatant in wells without cytopathic effect. It is important to highlight that although the IC_50_ values are lower than those reported by Jones et al. (2006), here we used human RBCs instead of chicken RBCs, which can affect the hemagglutination activity of viruses, since there are specific expression patterns in 2,3‐ and 2,6-linked sialic acid moieties on RBC surfaces (Eisfeld et al., 2014). In addition, we used different multiplicity of infection (MOI) amounts (0.001 vs. 0.01).

**Table 2:** Inhibition of viral replication with EB peptide.

Virus strain	Sample	IC_50_ (nM) Mean ± SD
A H1N1 pdm	EB MQ extract	20.71 ± 1.031
	WT MQ extract	0
	EB TRIS extract	21.25 ± 1.030
	WT TRIS extract	0
	EB Synthetic	754.4 ± 1.026

Concentration from 0 to 4000 nM of EB of transgenic microalgae extracts was analyzed for the ability to inhibit viral replication of 100 TCID_50_ of Influenza pdm A/H1N1. The results represent two independent assays. The IC_50_ and SD were determined for the dose-response curve.

## Discussion

Viruses have taken an unprecedented importance in Global Public Health, provoking human and economic losses and urging humanity to find solutions through the study of viruses, especially those that have a high mutation rate or a high transmission rate. The influenza virus is a pathogen that recurrently affects respiratory systems, and due to its special characteristics of genetic diversity mechanisms (such as rearrangement, recombination, and RNA polymerase errors), new resistant strains can arise (Webby et al., 2013). These situations thrust the importance of generating new antiviral drugs.

Currently, the most circulating influenza strains are resistant to adamantine and neuraminidase inhibitors. For this reason, other therapeutic targets are under investigation. One central point in the viral infection process is the binding of viral hemagglutinin to cell receptors. An excellent candidate to inhibit the binding is the EB peptide, by blocking the hemagglutinin-sialic acid interaction. In order to reduce production costs, we decided to express this peptide in *C. reinhardtii*. These microalgae have a great potential in biopharmaceuticals production, such as antibodies, growth factors, and enzymes because this platform production does not require a strict purification process, because it does not contain toxic elements for humans. This implies 80% cost reduction in the production process (Chen, 2008; Rosales-Mendoza et al., 2020).

*C. reinhardtii* has proven to be an effective platform for recombinant protein production reaching 0.02–2% TSP and a yield of 0.3–3 mg/L per culture medium in the case of expression in chloroplast (Rosales-Mendoza, 2013; Gimpel et al., 2015). Similarly, it is possible to express heterologous genes in the nuclear genome, but enhancing elements need to be used (Rasala et al., 2012). The EB peptide gene was codon optimized for expression in the nuclear genome of the microalgae. This avoids a depletion of the transcript because microalgae has an unusual use of codons with high GC content (>61%) and avoids heterochromatization (León and Galván, 2007). In the design of the EB peptide, the signal peptide Pr1a was added, which directs foreign proteins to the secretory pathway and, together with C-terminal KDEL sequence, prevents secretion of luminal ER proteins, therefore increasing the accumulation of recombinant proteins (Munro and Pelham, 1987; Sharma et al., 2000; Ziegler et al., 2000; Potvin and Zhang, 2010); this is an important approach to minimize foreign protein degradation by cytoplasmic proteases present in microalgae (Surzycki et al., 2009), since it keeps the recombinant protein bound to receptors into the inner membrane reticulum. Hempel et al. (2011) and Hempel and Maier (2012) demonstrated that there is an accumulation in the endoplasmic reticulum of *Phaeodactylum tricornatum* of an antibody against hepatitis B when an ER retention tag is added, and it prevented the export to the culture medium.

The maximum concentration of the EB peptide was obtained in aqueous extract reaching a yield of 4.99% of TSP. This high value is due to the endogenous proteins having less capacity to dissolve in water and remaining in the insoluble fraction. Therefore, the extraction microenvironment allows the EB peptide to have more access to the solvent; thus, the amount of EB is higher vs. endogenous proteins. The EB peptide yield represents 0.19% of dry biomass, which is higher than other biopharmaceutical products produced in microalgae. For example, Barahimipour et al. (2016) expressed the HIV antigen (P24) under the HSP70/RBCS2 fused promoter by nuclear transformation, and the yield was 0.25% of TSP (Barahimipour et al., 2016). The expression of erythropoietin has also been achieved with lower yield than in our work, rendering up to 0.03% of dry weight, in a work that includes the use of RBCS2 introns, which increase the transgene expression because the introns promote mRNA secondary structures causing higher efficiency of RNA polymerase II and longer messenger half-life (Eichler-Stahlberg et al., 2009). The differences in performance of recombinant protein production are not exclusive to microalgae, because protein accumulation is linked to stability of the messengers and the efficiency in the translation of mRNAs (Rasala et al., 2010).

Microalgae consumption in rats and humans has not shown toxicity; nevertheless, they have shown multiple benefits for gastrointestinal health (Tran et al., 2013; Endres and Murbach, 2018; Fields et al., 2020). Chávez et al. (2020) demonstrated that human pro-angiogenic growth factors (hVEGF-165, hPDGF-B, and hSDF-1) expressed in *C. reinhardtii* had the capacity of stimulating specific cellular processes without cytotoxic effect in mammalian cells. EB and WT extracts do not cause damage to MDCK cells; therefore, the EB peptide can be used as a prophylactic treatment to prevent viral spread in the respiratory tract.

Our results suggest that the EB peptide expressed in microalgae and also the synthetic peptide interact with viral hemagglutinin, avoiding binding with sialic acid site; therefore, the EB peptide prevents the first interaction between the virus and cell. Downard et al. (2016) reported that the EB peptide can stoichiometrically block the acid sialic binding sites. We observed that both peptides have wide protection ranges and a dose-dependent inhibitory effect, as reported by Jones et al. (2006). The effectiveness of the EB peptide expressed in microalgae is higher than the synthetic peptide against Pandemic and Virginia influenza strains. Different concentrations of the EB peptide are required for different viral strain inhibition. This effect was already reported by Jones et al. (2006), who reported that different glycosylation patterns of HA may have a slight effect in the interaction with EB. Therefore, additional experiments need to be performed for different influenza subtypes.

One goal of the antivirals is to prevent production of new infectious particles. The EB peptide’s efficiency to inhibit viral replication was demonstrated during the viral particle binding and internalization process (Downard et al., 2016). EB expressed in microalgae was able to inhibit viral replication with less amount than the synthetic peptide. This result is interesting because both peptides share the same functional consensus sequence RRKKLAVLLALLAP; they are hydrophobic amino acid clusters capable of interacting with the HA binding site (Bultmann et al., 2010; Jones et al., 2011a); however, the EB peptide expressed in microalgae has 10 amino acids more than the synthetic one (Enterokinase site and His-tag), which may provide more stability than the EB peptide alone. Another reason is that the synthetic EB peptide is highly hydrophobic and can render auto assembly (Bultmann et al., 2010), which compromises its bioavailability.

The soluble extract containing EB has an advantage regarding the synthetic peptide. This is the microenvironment of endogenous components of microalgae, which offers protection against degradation by adding trypsin in infection medium by virus processing; therefore, the peptide from microalgae water extract has an extended lifetime. The protective effect against mucosal protease degradation has been reported for biopharmaceuticals expressed in *C. reinhardtii* (Ochoa-Méndez et al., 2016; Carrizalez-López et al., 2018). Additionally, microalgae extracts have been shown to contain components enhancing biological activity, anti-inflammatory, and immunomodulatory properties (Rosales-Mendoza, 2013; Rosales-Mendoza et al., 2020). It will be necessary to prove the antiviral efficacy of EB peptide in an *in vivo* model.

*C. reinhardtii* has proven to be a good biopharmaceutical expression platform. We demonstrated that the EB peptide is expressed at high levels in transformed microalgae and can be extracted as part of total soluble fraction. We suggested that post-translational modifications conferred for microalgae to the EB peptide and the additional amino acids increased efficiency vs. the EB synthetic peptide. Our results show that microalgae as a platform can help us to improve the effectiveness of the EB peptide to inhibit flu virus replication. This promising result suggests that we can help to reduce the health burden caused by respiratory viral infections with antiviral peptides expressed in microalgae systems, which may reduce the spread of emerging viruses.

## Data Availability Statement

The original contributions presented in the study are included in the article/supplementary material; further inquiries can be directed to the corresponding author.

## Author Contributions

KR-B, AA-S, RS-G, LH, NS-J, CC-G, and RL-M designed the study. KR-B, LH, NS-J, and RL-M performed the experiments and collected the data. KR-B, AA-S, RS-G, LH, NS-J, RL-M, and CC-G analyzed, discussed, and interpreted the data. KR-B wrote the first manuscript and all co-authors contributed with substantive comments. LH and AA-S supervised the work and obtained the funding. All authors contributed to the article and approved the submitted version.

### Conflict of Interest

The authors declare that the research was conducted in the absence of any commercial or financial relationships that could be construed as a potential conflict of interest.
